# MagIO: Magnetic Field Strength Based Indoor- Outdoor Detection with a Commercial Smartphone

**DOI:** 10.3390/mi9100534

**Published:** 2018-10-20

**Authors:** Imran Ashraf, Soojung Hur, Yongwan Park

**Affiliations:** Department of Information and Communication Engineering, Yeungnam University, Gyeongsan, Gyeongbuk 38541, Korea; ashrafimran@live.com (I.A.); sjheo@ynu.ac.kr (S.H.)

**Keywords:** indoor-outdoor detection, smartphone sensors, magnetic field, context awareness, machine learning

## Abstract

A wide range of localization techniques has been proposed recently that leverage smartphone sensors. Context awareness serves as the backbone of these localization techniques, which helps them to shift the localization technologies to improve efficiency and energy utilization. Indoor-outdoor (IO) context sensing plays a vital role for such systems, which serve both indoor and outdoor localization. IO systems work with collaborative technologies including the Global Positioning System (GPS), cellular tower signals, Wi-Fi, Bluetooth and a variety of smartphone sensors. GPS- and Wi-Fi-based systems are power hungry, and their accuracy is severed by limiting factors like multipath, shadowing, etc. On the other hand, various built-in smartphone sensors can be deployed for environmental sensing. Although these sensors can play a crucial role, yet they are very less studied. This research aims at investigating the use of ambient magnetic field data alone from a smartphone for IO detection. The research first investigates the feasibility of utilizing magnetic field data alone for IO detection and then extracts different features suitable for IO detection to be used in machine learning-based classifiers to discriminate between indoor and outdoor environments. The experiments are performed at three different places including a subway station, a shopping mall and Yeungnam University (YU), Korea. The training data are collected from one spot of the campus, and testing is performed with data from various locations of the above-mentioned places. The experiment involves Samsung Galaxy S8, LG G6 and Samsung Galaxy Round smartphones. The results show that the magnetic data from smartphone magnetic sensor embody enough information and can discriminate the indoor environment from the outdoor environment. Naive Bayes (NB) outperforms with a classification accuracy of 83.26%, as against Support vector machines (SVM), random induction (RI), gradient boosting machines (GBM), random forest (RF), k-nearest neighbor (kNN) and decision trees (DT), whose accuracies are 67.21%, 73.38%, 73.40%, 78.59%, 69.53% and 68.60%, respectively. kNN, SVM and DT do not perform well when noisy data are used for classification. Additionally, other dynamic scenarios affect the attitude of magnetic data and degrade the performance of SVM, RI and GBM. NB and RF prove to be more noise tolerant and environment adaptable and perform very well in dynamic scenarios. Keeping in view the performance of these classifiers, an ensemble-based stacking scheme is presented, which utilizes DT and RI as the base learners and naive Bayes as the ensemble classifier. This approach is able to achieve an accuracy of 85.30% using the magnetic data of the smartphone magnetic sensor. Moreover, with an increase in training data, the accuracy of the stacking scheme can be elevated by 0.83%. The performance of the proposed approach is compared with GPS-, Wi-Fi- and light sensor-based IO detection.

## 1. Introduction

The hallmark of this decade is the wide proliferation of modern mobile devices that comprise mainly smartphones. According to World Bank statistics, the amount of current users of mobile devices is 4.57 billion, and this is expected to grow to 4.78 billion by 2020 [1]. These mobile devices provide hundreds of millions of apps (application) to assist the users to perform everyday activities. The extensive use of smartphones over the globe incited a new paradigm of services collectively called location-based services (LBS), which rely heavily on the location information. The field of LBS has received a rapid attention due to its pivotal role in many areas including tracking, providing on-the-go services in the proximity, navigation, location-sensitive billing and advertising and safety-related services [2]. LBS are provided through mobile apps depending on the user location, which is calculated through mobile devices and communication networks [3,4]. The LBS sector is gaining incremental demand and continual significant research attention from industry and academia alike.

One potential element that enables the efficient functioning of LBS is the indoor positioning and localization. Indoor positioning not only helps the customers in guiding them through departments to a particular product counter, it saves many lives in emergency response operations and navigates people to safety, as well. In addition, LBS serve a key role in other industries including tourism, intelligent transportation, gaming, social networking, etc. [5]. Many of the services provided by LBS are context aware. Context is defined as any information that can help in characterizing the current situation of an individual or thing, e.g., name, time, device, environment or location [6,7]. Context awareness helps many indoor positioning systems (IPS) work smoothly. For example, detecting whether a mobile user is indoors or outdoors can assist the IPS to switch sensors used for positioning and can increase energy efficiency, as well as the accuracy. Similarly, other personalized apps can use this information to set the preferences for the user accordingly, with respect to the sensed environment.

The indoor-outdoor (IO) environment sensing techniques are broadly classified under two categories: Global Positioning System (GPS) based techniques and smartphone sensor-based techniques.

### 1.1. GPS-Based IO Detection

GPS is a very reliable technology that serves as a de facto positioning technology in the outdoors. GPS-based IO detection exploits trilateration techniques, which require more than one satellite to achieve high positioning accuracy. In the open outdoor environments, usually, many satellites are available, so the positioning is accurate enough; sometimes to a few centimeters [8]. However, inside buildings, the satellite signals are absorbed or attenuated due to blocking ceilings and walls. Hence, the accuracy is severely degraded. In the situations where the user is close to windows, however, positioning can be performed, but in many cases, the error is larger than the indoor building area itself. This degradation in GPS signals is used as the main parameter that decides the user state of being indoors or outdoors for GPS-based IO detection. When the user enters a building, there is a sufficient drop in the signal’s strength, which can be used to infer if the user is moving from or to the indoor environment.

GPS-based IO detectors have two potential problems that limit their capability to determine the environment. First, in many cases, the signal degradation is not enough to point out the change in user state. For example, [9,10] suggested that if the building has wide windows, GPS is still able to point to the user position just like it does in the outdoor environment, and the user IO state cannot be determined with confidence. In such cases, it can assist in positioning, but cannot work as an IO state classifier. The second drawback that is very pivotal regarding user IO state is its high power consumption. The works in [11,12] indicated that GPS is the most power hungry among the smartphone sensors. It is reported to utilize seven-times higher energy as compared to inertial measurement unit (IMU) sensors available on the smartphone. Additionally, [13] states that the GPS continues to report fixes for a period of 10 to 35 s after the transition has happened from the outdoor to the indoor environment.

### 1.2. Smartphone Sensor-Based Indoor-Outdoor Detection

The second category involves exploiting the built-in sensors of the smartphone to perform IO detection. Smartphone sensors are used to apply fixes to the GPS’ limitations, and GPS is used opportunistically to save power. The sensors used for IO detection include Wi-Fi, ambient light sensors, iBeacon, proximity sensors and magnetometers. These sensors are called MEMS (micro-electro-mechanical-system), which are very small in size and more reliable than before. Smartphones, nowadays, are equipped with a built-in ambient light sensor, which can determine the intensity level of the surrounding environment. Its main purpose is to optimize the brightness of the smartphone screen accordingly, which allows one to save battery [14,15,16]. Light intensity varies amply while moving from indoors to outdoors and vice versa. Thus, the various levels of light intensity are used as the main parameter to detect user IO state. The proximity sensor is used to assist the light sensor to decide if the change in light intensity is caused by any object hindering the light sensor or a change in the environment. Smartphones also have built-in Bluetooth chips (BLE from 2013 onward) that are low energy and can communicate within a short range. Their signal intensity is scattered randomly and reduces over distance [17,18]. For IO systems, BLEs are installed indoor at specific entrance points, and the change in the received signal strength (RSS) values can point to the user IO state. The magnetometer is another useful sensor that is used to complement the light sensor and GPS to determine IO state. It measures the magnetic values of a particular location and can give *x*, *y* and *z* direction magnetic intensity. The geomagnetic field is smooth in outdoor environments; however, ferromagnetic materials cause disturbances inside. The smooth geomagnetic field is disturbed indoors due to iron, steel and similar other structures, which results in reasonable variation in the indoor environment. The variance is calculated for a number of frames, and a thresholding value can be used to determine user IO state. The strength of cellular signals is yet another indicator that is used to identify the transition from indoors to outdoors and vice versa. Signal strength acquired from the tower is used for this purpose, as there is a substantial drop in signal strength while moving outdoors to indoors. However, occasionally, this transition is very slow, and data from the tower are acquired for a longer period of time, which increases energy consumption. Wi-Fi uses the same principle to detect the indoor-to-outdoor transition. Since Wi-Fi APs (access points) are deployed in the indoors, their visibility and signal strength are higher than those of the outdoor environment, which can be used as the basis for such systems. However, the APs are to be scanned, and this consumes more time and energy than using the MEMS sensors of the smartphone.

This research aims at using the smartphone magnetometer alone for IO detection. The contributions of this research work can be summarized as below:

1. A feasibility study of using the geomagnetic field (referred to as ’magnetic field’ in the rest of the paper) to detect the user IO state.
2. The performance appraisal of machine learning-based techniques to predict IO state with smartphone sensor data alone.
3. An ensemble-based classifier to perform IO environment classification using magnetic field data from a smartphone.

To the best of the authors’ knowledge, this is the first attempt to use a magnetic field alone for the purpose of indoor-outdoor environment detection. The rest of the paper is organized in the following manner. Section 2 gives a short background of the magnetic field with respect to its use in indoor localization. Section 3 describes the related research in IO detection. Section 4 shows the feasibility analysis of using the magnetic field for IO state recognition. Section 5 overviews the machine learning techniques used in the current study. Section 6 discusses the results, and the conclusion is drawn in Section 7.

## 2. An Insight on the Magnetic Field

The Earth’s magnetic field is a natural phenomenon, which is pervasive. It extends from the Earth’s outer core until it meets the solar wind. The magnetic attitude of the Earth is caused due to convection currents, which result from the movement of molten iron in Earth’s outer core. It runs perpendicular to Earth and makes the Magnetic North and South Pole like geographic poles. However, the Magnetic North pole is tilted by 11 degrees from the Geographic North Pole. The magnitude of the magnetic field at the Earth’s surface extends between 25 and 65 μT (Tesla). Since it is ubiquitous, it has been used recently for many indoor positioning and localization systems [19,20,21,22].

There are a few characteristics of magnetic field measurements that are important regarding their use in the current study. The magnitude of the magnetic field is very smooth over a restricted area; however, it is disturbed by the presence of ferromagnetic materials including iron, nickel, cobalt, permalloy or any other combination of these metals, e.g., concrete. This feature is very important as the indoor localization systems take advantage of this feature to identify various locations indoor. Additionally, IO systems [13,23] also use this feature to discriminate the indoor and outdoor environment. The magnetic field is mutated over time and space, and the World Magnetic Model (WMM), which is used to calculate the magnetic field value, is revised after five years [24]. Various mobile devices exhibit various magnetic attitudes depending on the sensitivity of magnetometer used in that device [19,21]. Additionally, changing the orientations of the device also leads to changed magnetic field strength. The magnetic field is represented by seven features including inclination (I), declination (D), the horizontal magnitude (H), the total magnitude (F) and magnetic *x*, *y* and *z*. In practice, *F* is used mostly, as *x*, *y* and *z* are employable when the device orientation attitude is fixed. Therefore, low discernibility is another characteristic that restricts the use of magnetic fields [19].

## 3. Related Work

The research focusing on indoor and outdoor localization is increasing exponentially especially those that utilize smartphone sensors [25,26,27,28,29]. Such localization systems mostly work with the assumption that the system clearly has the knowledge of the indoor and outdoor environment, which is hardly true in practice. The detection of indoors/outdoors is needed to make the smooth switching of modes in many localization systems. A few research efforts that aim at the detection of IO environment are discussed here.

Zhou et al. in [23], made use of smartphone sensors including the accelerometer, proximity, light sensor, cell tower RSS and magnetometer to distinguish between outdoor, semi-outdoor and indoor environments. The patterns of different signals are studied under various weather conditions and at different times of the day. The patterns are used to define thresholds for various signals, and those thresholds are then integrated using the hidden Markov model. Radu Valentin et al. in [13], introduced the concept of machine learning for the IO detection problem. The study considered both supervised and semi-supervised training methods for the said problem. The semi-supervised learning approach is pretty useful when enough labeled data are not available for training purposes. It can improve classification accuracy by considering the unlabeled data from unfamiliar environments. The concept of co-training is utilized, which can accomplish an accuracy of 92.33% for IO detection in comparison to GPS (75.23%), IO Detector (48.51%) and naive Bayes (81.29%). Data were collected for this study from smartphone sensors including light, proximity, magnetic, microphone, cell, Wi-Fi, battery thermometer and GPS.

Bhargava et al. in [30], proposed a scheme named SenseMe, which can sense environmental context, as well as the context-aware location. The system used the data from GPS in addition to smartphone sensors’ data including gyroscope, accelerometer and the Bluetooth module. Three different environments, indoor, outdoor and indoor-outdoor, were detected using the C4.5 classifier on data collected over a time span of 1 min. The proposed scheme was able to achieve a classification accuracy of 98.4%, 93% and 82% for indoor, outdoor and indoor-outdoor environments, respectively. Canovas et al. in [31], presented a binary classification technique that utilized the RSSI (received signal strength indicator) from 802.11 access points to identify a device’s indoor or outdoor state. RSSI features from different APs were fed as input to a weak learner to be trained on, during the offline phase. The proposed method was compared with the nearest neighbor and naive Bayes to show its performance.

Sung et al. in [32], proposed an outdoor-indoor environment detection technique, which was based on the chirp sound probe. A binary classification method was used to determine the indoor and outdoor environments by using the reverberation score, which was calculated with the help of the envelope of the sound. An empirical threshold value for indoor and outdoor environments was applied to the reverberation scores for classification. The proposed method achieved the transition detection in only 3.81 s on average. Liu et al. in [33], proposed a method to detect if a mobile phone user was currently in the indoor or outdoor environment. The proposed method was based on the cell identity map in addition to the light and proximity sensors of a mobile phone. The light sensor was used mainly for IO detection with a threshold value for both environments. However, in case the light sensor were absent, the cell identity map was utilized for the said purpose. The cell identity map was built based on the indoor deployed cells to increase the mobile broadband capacity in the 3G and 4G networks. Cell identity and its relevant RSS were used to identify the indoor and outdoor environment. The proposed scheme was able to achieve 98% accuracy.

Okamoto et al. in [34], presented a system that complements GPS-based IO detection by adding moving direction information. The GPS-based environment detection was done with support vector machines by utilizing the S/N ratio. It achieved an accuracy of 96%. Later, direction sensing was done with a compass, and environment sensing was performed for the canyon of the buildings. The method with direction information performed well compared to a GPS-based system in canyons. Bisio et al. in [35], presented a method based on the ultrasonic signals sensed by a smartphone. The phone’s built-in speakers and microphone were used to get the features required for IO classification. The features were then fed into a support vector machine and naive Bayes classifier for classification. The ultrasonic-based environment sensing system achieved an accuracy of 88.9% for fast latency and 92.7% for slow latency. An indoor-outdoor detection technique was presented by Zou et al. in [36], which leveraged the low power iBeacon technology. The environment was divided into outdoor, semi-outdoor and indoor classes. For the outdoor environment, GPS was used, which was turned off once the semi-outdoor state was confirmed, which was triggered by the decrease in mean GPS signals. iBeacon was utilized to discriminate between semi-outdoor and indoor environments. Two BLE beacons were installed at each entrance point, which marked the transitions between semi-outdoor and indoor environments. The user’s state of coming into and going from an indoor environment was established based on the RSS values of BLE beacons. The environment detection accuracy of the proposed scheme was 96.2%.

Wang et al. in [37], utilized the machine learning algorithm to identify the user’s context of being indoors and outdoors. The Global System for Mobile (GSM) communication cellular base station’s signal strength was exploited for that purpose. Since the signal strength was affected by various environments, the single strength characteristic was utilized. The data were collected at 2 Hz to classify four environments including deep indoor, semi-outdoor, light indoor and open outdoor environments. Machine learning algorithms of support vector machine, k nearest neighbor, decision trees, naive Bayes, logistic regression, nearest neighbor and random forest were tested on 8 s of data for this purpose. Random forest was reported to achieve the highest accuracy of 95.3% on the GSM data with four nearby satellites. He et al. in [38], presented the IO environment detection as a one-class inside-outside region detection. The radio map of Wi-Fi was built during the offline phase, which was later utilized in different machine learning techniques. Support vector data description, self-organizing map, mini-max probability machine and principal component analysis were used for classification. The measured signal was mapped against the pre-built database. If the measured signal resembled a fingerprint (indoor region), then it was highly likely to belong to the indoor region, and outdoor otherwise. Principal component analysis (PCA) achieved the highest accuracy of 95.69% for IO classification.

Li et al. in [39], presented a lightweight IO detector based on Wi-Fi RSS signals with a light sensor. The light sensor was assisted by the proximity sensor to validate if the light sensor were blocked by an obstacle. Various thresholds were used for the light sensor for day and night and for the indoor-outdoor environment. Wi-Fi RSS and the light sensor detected the environment separately, and their aggregate was used in a semi-CRF (conditional random field) algorithm to generate the integrated output. The proposed system achieved an accuracy of 96.67%.

The discussed research works were constrained by one or more limitations. For example, [23] made use of the variance of the magnetic component for data taken over 10 s. First, using the variance alone is not a good parameter, as the variance is abruptly affected in the outdoor environment as well, due to the presence of vehicles. Secondly, data were collected over 8 to 10 s, which consumed a substantial amount of energy. Similarly, IO systems that utilize cell tower data or GPS data have the conspicuous drawback of energy consumption. The systems based on iBeacon and Wi-Fi are susceptible to infrastructure changes and rely on additional hardware and software. We, therefore, focus on the magnetic field, which is both energy efficient and omnipresent and needs no additional infrastructure. In addition, we are working with magnetic field data collected over 2 s only, which is robust and energy efficient.

## 4. The Feasibility of Using the Magnetic Field for IO Detection

**Figure 1:**
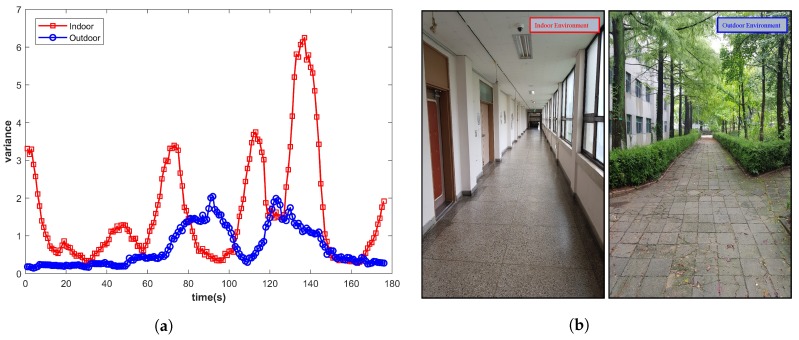
Magnetic variance for the indoor and outdoor environment. (**a**) Magnetic variance; (**b**) Indoor & outdoor environment of given variance.

The IO systems [13,23] use magnetic field strength as one of the components in their IO system, which assists in detecting the environment transition. They, however, rely on the variance of the magnetic field, which is not a good measure. We analyze the variance of various samples from indoor and outdoor environments to corroborate this, and the results are shown in Figure 1. Each plotted value shows the variance calculated over 2 s of data collected at a sampling rate of 10 Hz.

**Figure 2:**
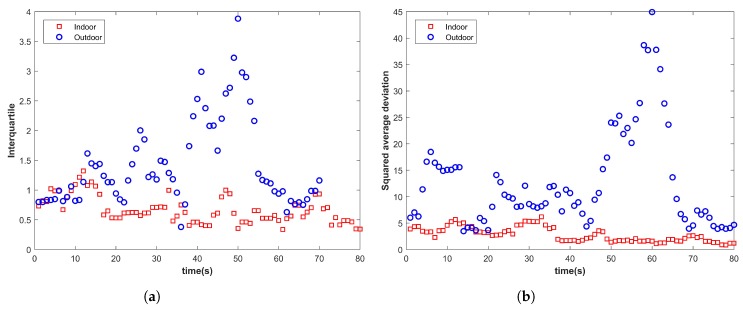
Magnetic features for the indoor and outdoor environment. (**a**) Inter-quartile; (**b**) Squared average deviation.

As shown in Figure 1, many indoor and outdoor environments demonstrate similar variance values. Since the magnetic variance depends on the interference caused by the infrastructure, similar indoor infrastructure may lead to very similar magnetic values, which ultimately results in small variance resembling the outdoor environment. We identify and categorize magnetic features into two groups. The first group of features is self-sufficient and individually unique for the IO environment, while the features in the second group are not individually characteristic of any environment, yet can discriminate IO environments when joined with other features. The first group, for example, includes coefficient, inter-quartile, squared average deviation, etc., as shown in Figure 2.

**Figure 3:**
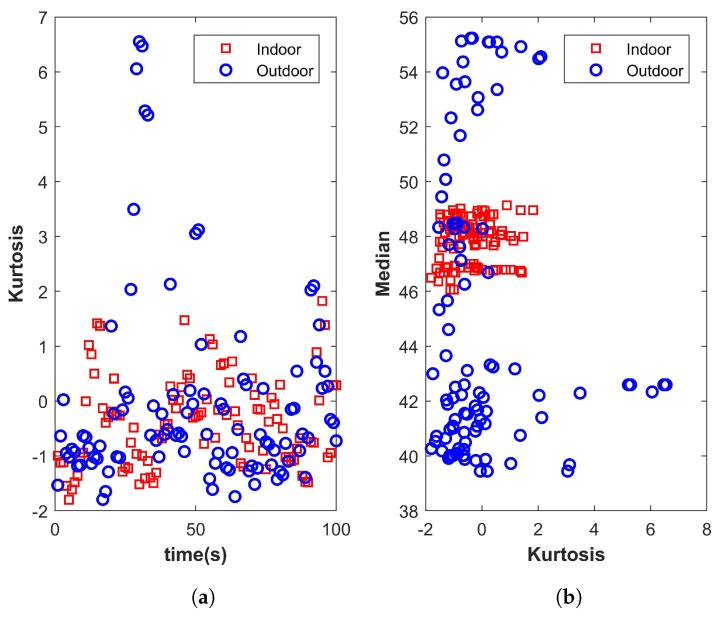
(**a**) Kurtosis and (**b**) kurtosis and median for the indoor and outdoor environment.

Figure 3 shows the plots for kurtosis, and it shows that it is not a good indicator to identify the indoor and outdoor environment. However, when plotted against the median for the same data, it becomes more meaningful and definitive.

The biggest challenge to use magnetic data for IO detection is its low dimension. The magnetic field is represented by seven characteristics, but only four of them are used including magnetic values in the *x*, *y*, *z* directions and total intensity *F*. Magnetic *x*, *y*, and *z* change with the change in phone orientation, and a fixed orientation is required if we want to use them. Limiting the user to always hold the phone in navigation mode is not always practical. Therefore, only the total magnetic intensity *F* can be used if we do not want to restrict the user. Additionally, total magnetic intensity *F* is not good enough for IO detection, as *F* can be very similar for many indoor and outdoor scenes. Such low dimensional data necessitate finding extended features that can help to determine the user IO state. During our feasibility study, we extracted 16 different spatial features, which can be used to distinguish between indoor and outdoor environments, individually or collectively. Table 1 shows all spatial features for this analysis.

Magnetic data have been used in many research works [19,20,21,25,26] for localization, yet this is first attempt to build an IO system based on magnetic data alone. It is not very practical and efficient to use a simple threshold on the extracted magnetic features as done previously [23]. Machine learning techniques are very useful to understand the data features and infer decisions. The following section discusses the most commonly-used machine learning techniques for classification that we intend to use on magnetic data for IO detection.

## 5. Machine Learning Techniques Used for Classification

We consider the IO detection as a classification problem with respect to machine learning where given a set of features, classification can be performed. The machine learning techniques are categorized into supervised and unsupervised depending on the method used during the training phase. Unsupervised learning is more useful when we do not have labeled data; based on the features learned from the training data, it can cluster the data into different classes. Supervised learning, on the other hand, takes the data with labels for various sets of features during the training phase to train the classifier. Later, the new data can be fed into the classifier to perform classification. The supervised learning classifier can infer complex relations and is more powerful than those who treat the features separately. The techniques used for the experiments are discussed here briefly.

### 5.1. Decision Trees

The decision trees are one of the most popular algorithms of machine learning, which can imitate the human decision making process. They are a simple yet powerful tool to understand the features and infer decisions. A decision tree [40] is composed of three types of nodes: the root node with no incoming edges and zero or more outgoing edges, the internal node with exactly one incoming edge and two or more outgoing edges and the leaf/terminal with exactly one incoming edge and no outgoing node. Each node denotes a feature, while each edge/link is a decision/rule [41]. Every node is followed by an attribute $a^{i}$. The tree can either be binary or non-binary; where the binary tree has exactly two leaves of a node, while node leaves in a non-binary tree depend on the number of elements of $A^{i}$ [42]. The gain ratio is used as the splitting criteria to determine the goodness of a split:

(1) $$\mathrm{Gain}\;\mathrm{ratio}=\frac{\Delta_{info}}{\mathrm{Split}\;\mathrm{Info}}$$

where,

(2) $$\mathrm{Split}\;\mathrm{Info}=-\sum_{i=1}^{k}P\left(v_{i}\right)log_{2}P\left(v_{i}\right)$$

**Figure 4:**
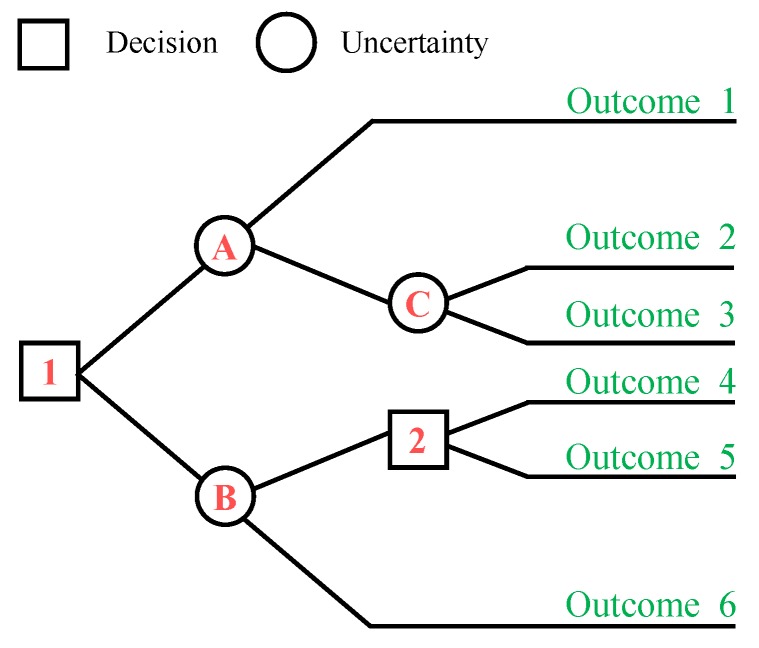
Decision tree.

*K* represents the total number of splits, which is set to four in the current study. The tree-pruning step is performed as well in order to reduce the size of the tree. Decision trees are favorable due to many reasons. First, they are non-parametric and do not require any prior assumptions about the data. Second, they are computationally inexpensive and easy to interpret, especially in the case of small trees. Last but not least, the redundant attributes in the data do not affect the accuracy of decision trees. A typical decision tree is shown in Figure 4.

### 5.2. k-Nearest Neighbor

**Figure 5:**
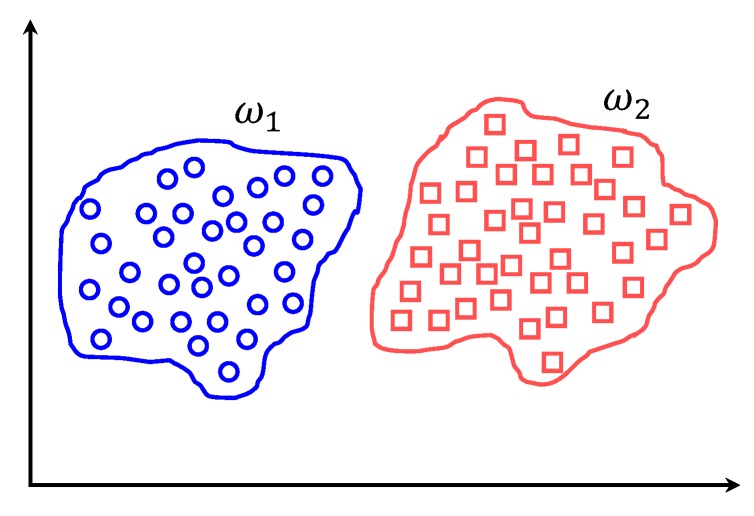
k nearest neighbor.

The nearest neighbor rule is one of the widely-used classifiers, which is known for its simplicity and efficiency. It is a non-parametric technique that does not make any assumptions on the underlying data distribution. It is often called a lazy learner as it considers the training examples as the classifier during its training phase. kNN derives a boundary between different classes as shown in Figure 5 considering the given number of *k* neighbors.

For example, for given data points:

$$\left\{\begin{array}{c} x_{11},x_{12},x_{13},\ldots ,x_{1m},y_{1}\\ \ldots \\ x_{n1},x_{n2},x_{n3},\ldots ,x_{nm},y_{n} \end{array}\right\}$$

where *x* shows the attributes used to predict the class (target) *y*. The distance function is defined to calculate the distance between the data points to form the boundaries between classes.

Often, Euclidean distance is a good choice if the data points are numerical. The odd values of *k* are selected in order to break the ties, and larger *k* values may help to overcome the adverse effect of noisy data on accuracy. A new object is classified to a class based on the majority voting of its neighbors [43,44]. For example, if the attributes of a new data point are $a_{1}$ to $a_{m}$, it can be associated with a particular class based on the distance calculated between points to any $x_{j}$ using:

(3) $$d\left(a,x_{j}\right)=\sqrt{{\left(a_{1}-x_{j1}\right)}^{2}+\ldots +{\left(a_{m}-x_{jm}\right)}^{2}}$$

Although simple and easy to interpret, kNN is computationally expensive due to the higher memory requirement, as it stores almost all training data. The prediction stage may become slow in case of big *N*, and it is sensitive to irrelevant features, as well.

### 5.3. Naive Bayes

The naive Bayes classifier, which is based on the Bayesian theorem, is a statistical classifier that can predict the probability of a given sample to a particular class. In spite of its simplicity, often, naive Bayes is very effective and can often outperform many sophisticated classification methods [45,46,47]. It calculates the posteriori probability $P\left(H|X\right)$, which is the probability that the hypothesis *H* holds given the observed data sample *X*. Let $X=\{x_{1},x_{2},\ldots ,x_{n}\}$ be a training set of samples with *n* attributes $A_{1},A_{2},\ldots ,A_{n}$ and having class labels $C=\{c_{1},c_{2},\ldots ,c_{n}\}$; according to Bayes rule, $P\left(C|X\right)$ can be expressed as:

(4) $$P\left(C_{i}|X\right)=\frac{P\left(X|C_{i}\right)P\left(C_{i}\right)}{P\left(X\right)}$$

Naive Bayes assumes that the values of attributes are conditionally independent of one another, which means that:

(5) $$P\left(X|C_{i}\right)\approx \prod_{k=1}^{n}P\left(x_{k}|C_{i}\right)$$

Therefore, given a new sample *x*, the class $C_{j}$ is given to the sample that achieves the highest posterior probability.

### 5.4. Random Forest

**Figure 6:**
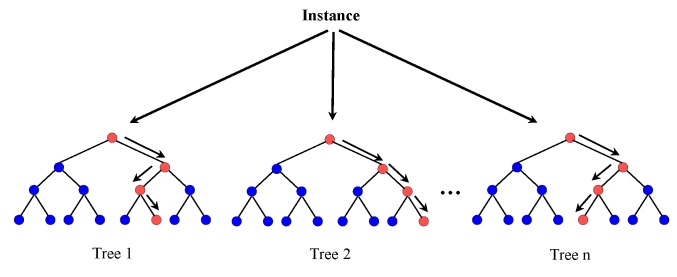
Random forest.

Random forest is a combination of tree classifiers where each tree is generated with a random vector from the input vector. Each tree has a unit vote for the most probable class to whom the input vector belongs [48]. A typical random forest is shown in Figure 6.

Random forest possesses the capability to handle datasets that are sparse, contain errors, as well as having missing values [49]. The design of a decision tree involves an attribute selection measure and a pruning measure. The information gain ratio criterion [50,51] and Gini index are the two most commonly-used attribute selection measures. We have used the information gain measure for our experiment. The number of features used and the number of trees are two parameters defined by the user. The maximal depth is set to 20, the confidence level to 0.25, a minimal gain of 0.1, a minimal leaf size of 2, a minimal size for the split to 4 and the number of pre-pruning alternatives set to 3 in our experiment.

### 5.5. Gradient Boosting Machines

**Figure 7:**
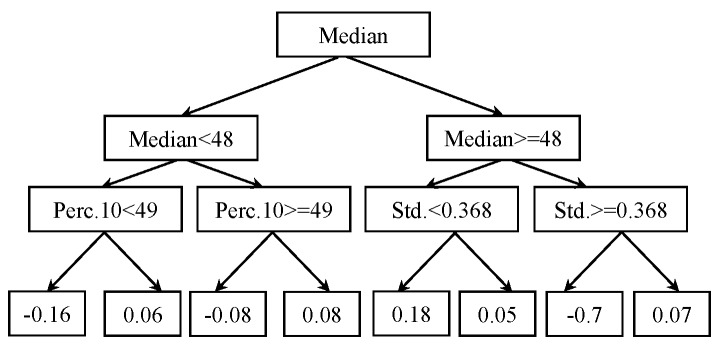
Gradient boosting machine.

Gradient boosting is a technique used in regression and classification problems, and it generates a prediction model in the form of decision trees. It has exhibited substantial success for practical applications in machine learning challenges [52,53]. The gradient descent method is selected as the loss function and minimized.

(6) $$y_{i}^{p}=y_{i}^{p}-\alpha *\sum \left(y_{i}-y_{i}^{p}\right)$$

where α is the learning rate, which is set to 0.1 in this experiment, and $\sum \left(y_{i}-y_{i}^{p}\right)$ is the sum of the residuals. The number of trees is set to 20, and the Bernoulli distribution is used. A gradient boosting machine is shown in Figure 7.

### 5.6. Rule Induction

Rule induction produces classifiers that are based on simple, yet effective and powerful ‘If–Then’ rules for decision makers [54]. The data contain regularities in the form of attributes, which are frequently expressed in terms of rules [55], so those rules can be written and used to describe the relation:

If *(attribute-1, value-1) and (attribute-2, value-2) and (attribute-n, value-n) then (decision, value)*.

**Figure 8:**
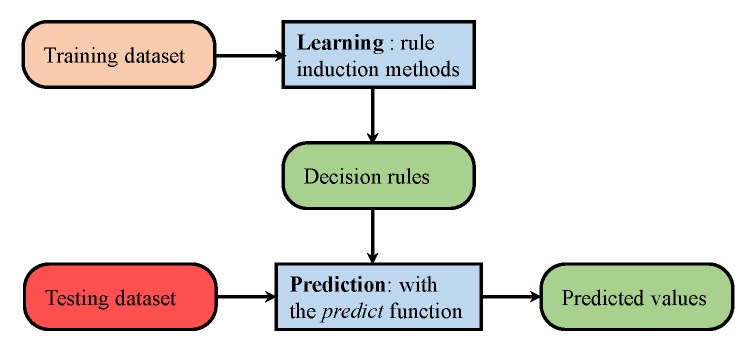
Rule induction.

Rule induction systems may contain simple or more complex rules depending on the attributes of the data. A rule induction system with simple rules is shown in Figure 8. The RIPPER (repeated incremental pruning to produce error reduction) algorithm is used for the current rule induction classifier. For our two-class problem, one class is chosen as the positive class, and an incremental pruning is done [56,57]. Information gain is used as a criterion for attribute selection and numerical splits.

### 5.7. Support Vector Machines

**Figure 9:**
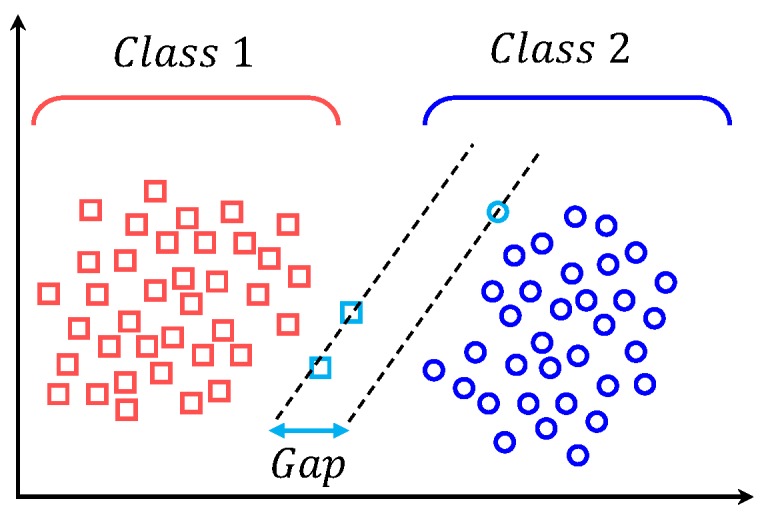
Support vector machine.

Support vector machines were developed by Cortes and Vapnik [58] for binary classification. SVM represent a powerful machine learning technique, which can perform nonlinear classification, regression and outlier detection [59]. SVM perform class separation by dividing the hyperplane optimally such that the distance/gap between the two classes is maximized. The points lying on the boundaries are called support vectors, as shown in Figure 9.

The higher the distance between these support vectors is, the more distinctive the classes are. The hyperplane parameters are derived from a quadratic programming optimization problem [60]. Various kernel types are used in SVM, including radial, polynomial, neural, etc.; we use the Epanechnikov kernel [61] for our experiment.

## 6. Experiment and Results

This section describes the experimental setup and the data collection methodology, as well as the experiment location. It also includes the analysis of the results from the selected classifiers.

### 6.1. Experimental Setup

**Figure 10:**
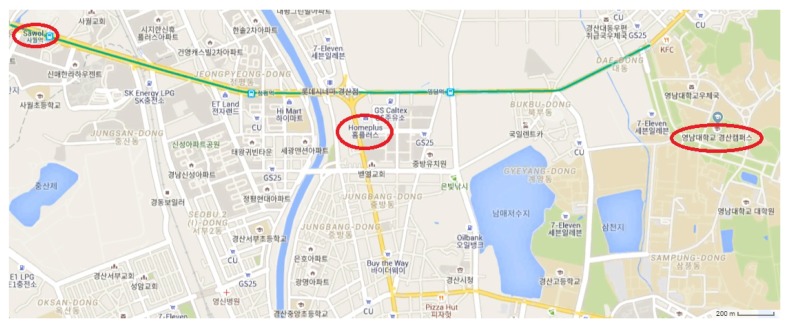
Experiment places.

The experiments are performed at three different places (as shown in Figure 10) including Yeungnam University, Gyeongsan campus, a shopping mall and an underground subway station in the Republic of Korea. The data are collected from various parts of the campus for indoor and outdoor environments. The shopping mall (Homeplus) is a 6-floor building including 2 basements. Indoor data are collected from all floors, while outdoor data are collected from its surroundings. The mall is situated on the brink of the main road, and heavy traffic makes magnetic data volatile.

**Figure 11:**
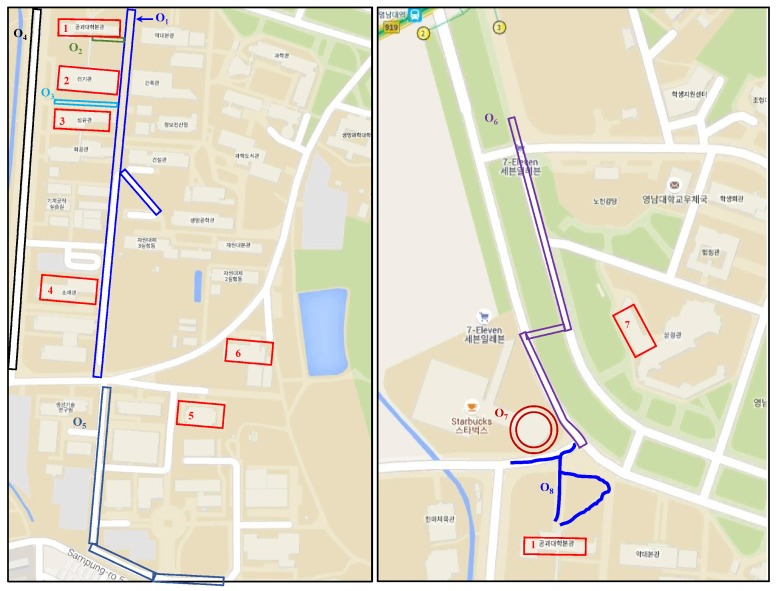
Indoor and outdoor area for the experiment.

Figure 11 shows the campus areas where the experiment is performed, as well as different walking traces followed during the outdoor data collection. A unique name is used for each trace as we want to analyze the result of each walking trace for a detailed analysis.

**Figure 12:**
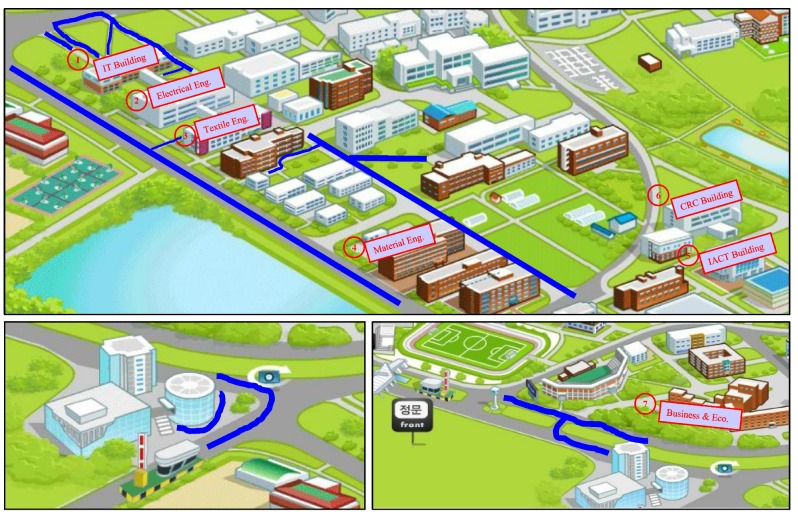
Walking paths for indoor and outdoor on the campus.

Figure 12 highlights the buildings that are used for the indoor environment experiment. The red outlined buildings include those where the data are collected for indoor scenarios, while the outdoor environments (in blue) include open roads, as well as small paths between the departments. It is important to describe here how we attribute an environment as indoors or outdoors, as it may be different considering that the magnetic field behaves differently than those of Wi-Fi, GPS, cellular tower signals, etc.

The data from the underground subway are collected from the entrance and exit points where we have enough space, as well as the waiting area. We did not collect data from the platform area as the transportation of the metro affects the magnetic data.

**Figure 13:**
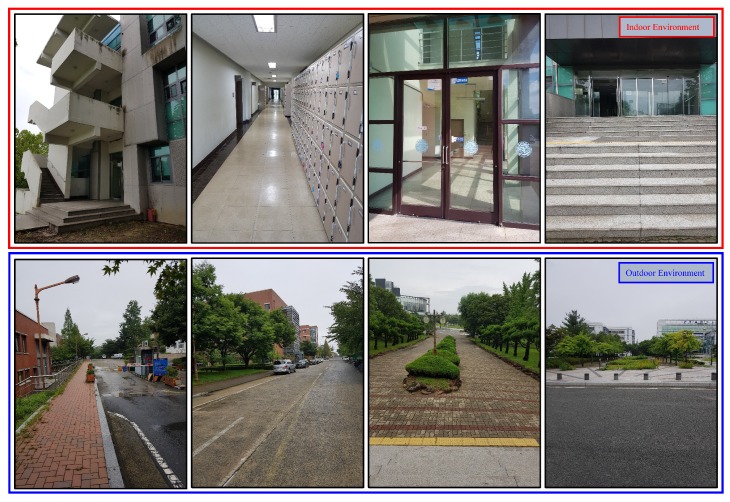
Indoor and outdoor scenarios.

Figure 13 shows various scenes that are included in indoor and outdoor environments. The covered entrances of the buildings, as well as the stairs connected with the buildings are categorized as the indoor environment. The rationale to associate them with the indoor environment is the impact that indoor infrastructure has on the magnetic field. As mentioned earlier, the magnetic field is disrupted due to ferromagnetic materials; the stairs connected with the buildings and entrances have similar perturbations. Therefore, the main entrances and similar other structures are classified as indoors. However, open areas including the ones that are even very close to the buildings are categorized as outdoors.

### 6.2. Data Collection

The data are collected using a Samsung Galaxy S8 smartphone, which has the Octa-core QUALCOMM Snapdragon 835 chip and Adreno 540 GPU. It operates on Android 7.0 Nougat with 4 GB RAM (random access memory). It is equipped with the Asahi Kasei Micro device AK09916C, which is a 3-axis magnetometer device. The device operates on 16-bit data for each 3-axis magnetometer component. The device sensitivity is 0.15 μT/LSB (Least Significant Bit), and the operating temperature is −30 °C to +85 °C. The operating supply voltage is +1.65 V to +1.95 V, and average current consumption at a 100-Hz repetition rate is 1.1 mA [62].

The data for the current study are collected at 10 Hz using a smartphone application designed in Android Studio 2.3.3. The data need to be collected while the user is walking. The features are gathered by processing 20 samples (2 s of data at 10 Hz) of magnetic total intensity *F*. The smartphone magnetometer returns magnetic field values $m_{x}$, $m_{y}$ and $m_{z}$ for the *x*, *y* and *z* axis respectively, which are used to get *F* using:

(7) $$F=\sqrt{m_{x}^{2}+m_{y}^{2}+m_{z}^{2}}$$

The testing is performed with three smartphones including the Samsung Galaxy S8 (SM-G950) (Samsung, Seoul, Korea), LG G6 (LGM-G600L) (LG, Seoul, Korea) and Samsung Galaxy Round (SM-G910S) (Samsung). The purpose of using various smartphones is to check the general applicability of the proposed approach. It is also noteworthy to mention that the smartphone orientation is traditionally fixed in localization systems that use magnetic data. We, on the other hand, allow the user to walk in any direction with any phone orientation including in the hand, call listening, in the pocket, etc.

### 6.3. Results

The extracted features were fed into the selected machine learning algorithms to classify the magnetic data samples taken from the indoor and outdoor environment. The accuracy measure was used to evaluate the performance of selected algorithms using the following equation:

(8) $$Accuracy=\frac{TP+TN}{TP+TN+FP+FN}\times 100$$

where *TP*, *TN*, *FP* and *FN* represent the true positive, true negative, false positive and false negative labeled by the classifier. Accuracy is a very widely-used measure, which defines the performance of a classifier in terms of correct prediction [63].

**Figure 14:**
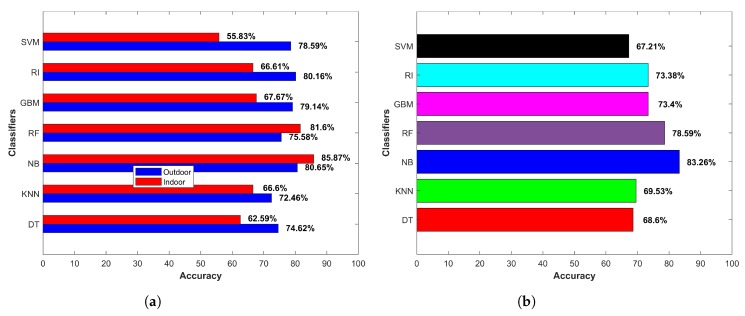
(**a**) Indoor-outdoor accuracy and (**b**) average accuracy.

Figure 14a shows the accuracy for indoor and outdoor cases separately, while Figure 14b indicates the averaged accuracy over both cases. The results demonstrate that the smartphone sensors’ data could potentially be used to extract features to be used in the classifiers for IO classification. One important point though to consider is that the provided data and its relevant features should be representative of the data pertaining to the areas where prediction was to be done. It is noteworthy to remark here that we did not use *k*-fold cross-validation. The results from cross-validation were deceptive as the cross-validation involved taking the labeled data of all environments where they were to be tested.

The training data for an indoor environment were taken from IT building Floor 3 alone. The testing was performed on the data collected from six buildings of the campus, as well as a subway station, and a shopping mall with four floors and two basements. Similarly, the outdoor data were collected at one point O${}_{2}$, shown in Figure 11, and data collected from various areas of the campus and two other locations were used for the testing. The purpose was to verify whether the selected features and classifier could show similar performance when extracted features were from the data of other similar environments. The results given in Figure 14 shows that the selected classifier performed well. DT, NB and RF performed better in the outdoor environment, with NB surpassing other classifiers with an accuracy of 86.35% for the outdoor environments. SVM, RF, gradient boosting machine (GBM) and kNN performed better in the indoor environment; however, NB superseded them with an accuracy of 82.05% for classifying the data from the indoor environments.

**Figure 15:**
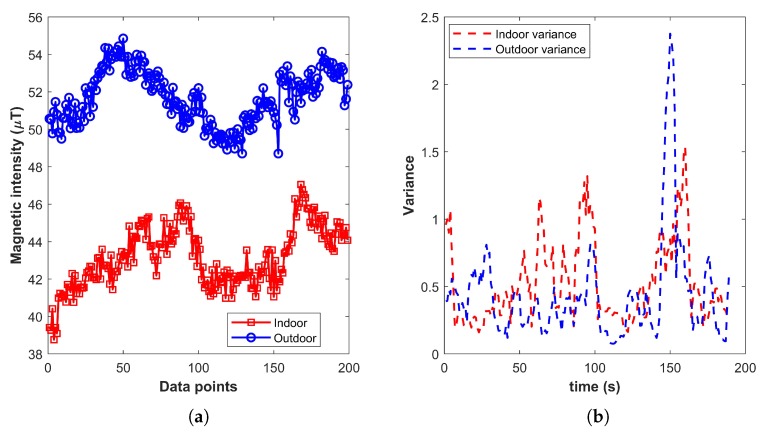
(**a**) Indoor-outdoor total magnetic intensity and (**b**) magnetic variance.

We further analyzed the results to find out the root causes of a few classifiers’ poor performances. We found that in two scenarios specifically, all classifiers except NB and RF showed poor performance. Consider the graph shown in Figure 15, which is for an indoor and outdoor instance. The magnetic field values for both cases behaved in a similar manner, and it was very hard to discriminate between the scenarios. In many buildings, when the infrastructure was very similar, there was much less or a very smooth change in the magnetic field. Such indoor environments’ magnetic attitude makes it very difficult to distinguish them from outdoor environments. The graph in Figure 15 does not show IO characteristic data, rather it shows anomalous behavior. Traditionally, outdoor data are smoother while indoor magnetic data show variations. Although the magnetic intensity was different for both cases, we were not using magnetic intensity for classification as it can be very similar for indoor and outdoor environments. Variance for given magnetic data was also very similar, and so for other features that were used for classification. Such uncommon magnetic data reduced the classification accuracy.

DT, SVM, GBM, rule induction (RI) and kNN classification degraded severely, and their classification accuracy went below 50%. However, NB and RF performed well even for such diverse cases. The outdoor plot was for the parking area in front of the IT building, which though outdoors, showed a magnetic attitude similar to the indoor environment.

**Figure 16:**
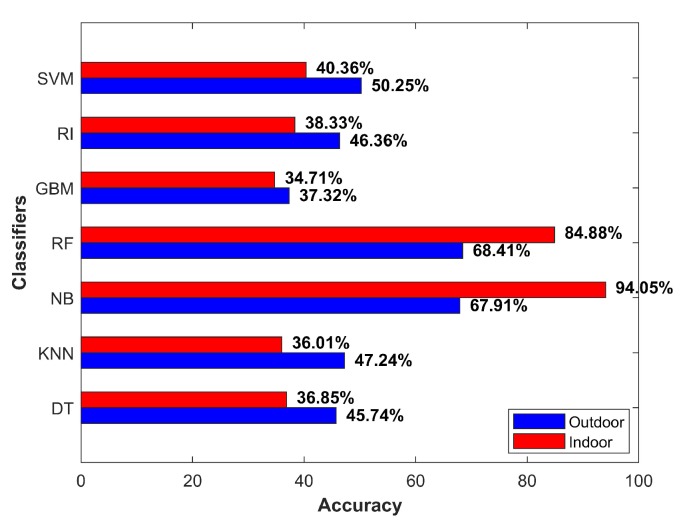
Accuracy for worst indoor and outdoor scenarios.

As mentioned earlier, the ferromagnetic materials intercept the magnetic field strength and cause anomalies. The presence of vehicles in the parking area generated the same effect provided the magnetic sensor came within a proximity of 1 m. Such disturbances led to sudden variance in magnetic field values and caused classification errors. Additionally, the noise in the data also affected the accuracy. Figure 16 shows the accuracy results for the above-discussed scenarios.

**Figure 17:**
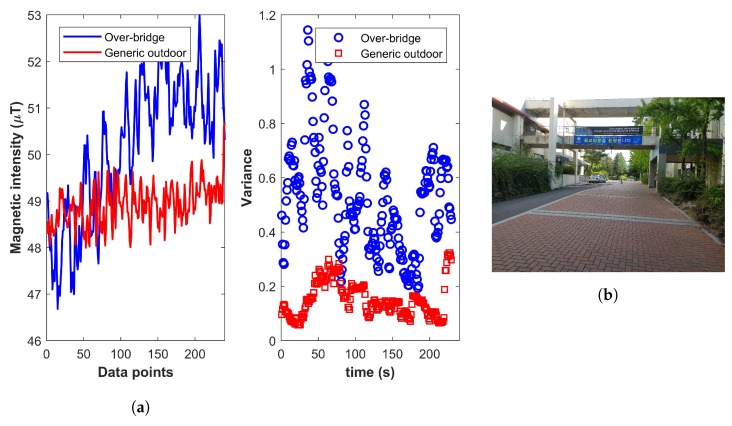
(**a**) Magnetic field intensity and variance for the over-bridge in the outdoors and (**b**) an over-bridge between two departments.

**Figure 18:**
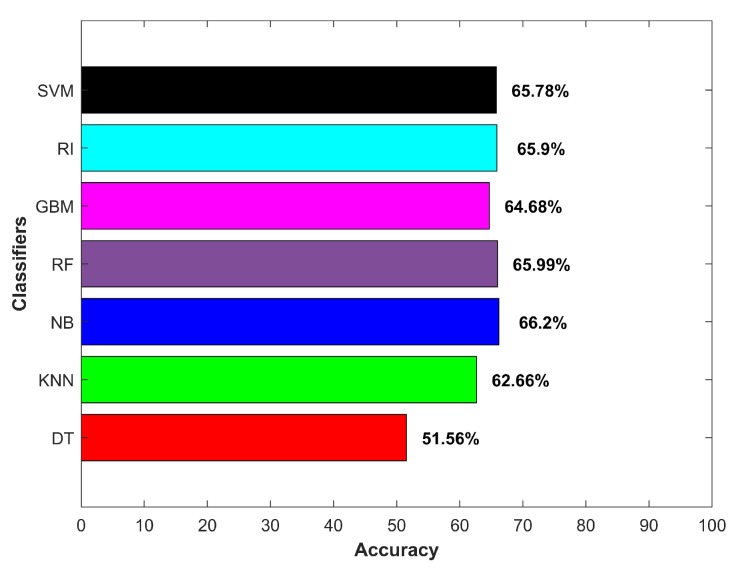
Classifiers accuracy for an over-bridge in the outdoors.

The second scenario that affected accuracy was an over-bridge of concrete, connected to main buildings on both sides. The magnetic field attitude was affected when walking under the bridge in a similar fashion as it did in the indoor environment. Consequently, the magnetic field intensity was very similar to the indoor environment at some places, which ultimately affected the extracted features, as well. Figure 17 shows the magnetic field value and resulting variance of a generic outdoor environment and data taken under the over-bridge. Since we used the extended features, which were actually based on only one attribute (magnetic field intensity), the magnetic field strength ultimately led to poor features. Hence, the performance of all classifiers was affected in this scenario, as shown in Figure 18.

The given two scenarios were very challenging; however, over-bridges are not common. Moreover, the results were only affected when such bridges were concrete and specifically situated between two closely-located buildings. Another important point is that the classification was based on magnetic field data alone, and the sample was only taken for 2 s, which is a very short time as compared to the 10 s used in [13,23]. The results demonstrate that machine learning algorithms were good enough to perform the classification with magnetic features only. Based on our findings, we worked out a classification model based on ensemble learning.

Ensemble learning methods leverage the outputs of the individual classifiers to make predictions for complex tasks [64]. Ensemble approaches tend to show better predictive performance than those of individual models [65]. Ensemble methods are generally categorized into two groups: sequential ensembles and parallel ensembles. Bagging and boosting are two popular sequential ensembles that utilize homogeneous ensembles. The motivation is to exploit the dependence between the base learners to improve the performance [66,67]. However, homogeneous ensembles are not a good choice when the base classifier is unclear. Therefore, heterogeneous ensembles are more appropriate and model the independence between the base learners. Stacking and ensemble selection are two very famous heterogeneous ensembles, which are based on meta-learning [68,69]. Stacking constructs a higher level classifier formulated over one or more base learner classifiers. Stacking is superior to single model classification, as it can utilize multiple heterogeneous classifiers to form a base model and then use it to make the final classification [70].

**Figure 19:**
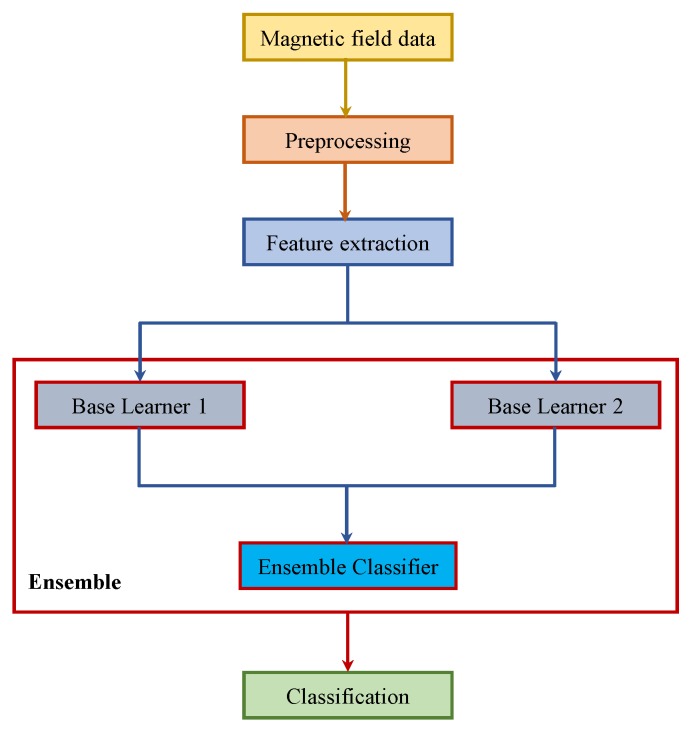
Stacking methodology used.

Figure 19 shows the diagram of the stacking approach for indoor-outdoor classification. We used RI and DT as the base learners, and their models were fed to NB to perform the final classification. Pre-processing was done in order to remove the noise from the data with a moving mean window of five samples. The training and testing procedure was the same as previously described. Indoor data from IT building and outdoor data from O${}_{2}$ were used for the training, while the data from other environments were used for testing purposes.

**Figure 20:**
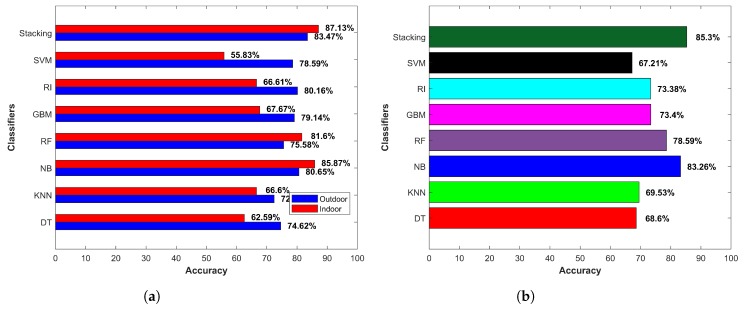
(**a**) Indoor-outdoor accuracy and (**b**) average accuracy.

Results for indoor and outdoor accuracy, as well as average accuracy are shown in Figure 20. Stacking outperformed other single classifiers and achieved an accuracy of 85.30%, which was higher than that of Naive Bayes alone. As other research [13,31,37,38] states that combining weak learners as the base learners and using their learned models as an input to a higher class model helps to improve the performance, we obtained similar results. However, the improvement in the performance was not much higher as compared to NB. The primary reason is that the basic attribute that was used to get extended features was only one, and extended features depended solely on that attribute. Even so, the achieved accuracy was comparable to the pioneer works [13,23] on IO detection, which utilized a variety of sensors including GPS, light sensor, Wi-Fi, cellular tower signal strength and magnetometer combined and achieved an accuracy of 92.33%.

**Figure 21:**
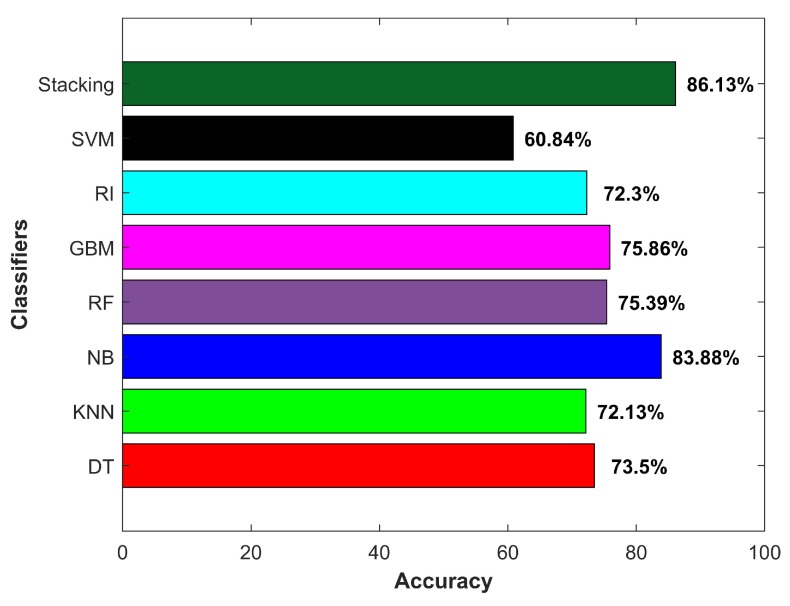
Indoor-outdoor accuracy with increased training data.

The performance of the machine learning classifiers was good considering the fact that only a small amount of training data had been utilized. One important and interesting aspect would be to consider the use of an increased amount of data for training. There are two possibilities for this consideration. First is to use the training data from each location where the experiment is performed. This option is, however, very impractical because in real-world scenarios, it is not possible to collect the data from every location where we intend to use the system for IO detection. Additionally, there are always unfamiliar environments, and a generalized system should be adaptable to such dynamic scenarios. The second option is to use more data from the location that was already used to collect the training data. We adopted the second option and increased the amount of training data by 1.5-times the data previously used. The results are shown in Figure 21.

The accuracy of SVM was affected adversely, while the performance of RI and RF was reduced a little. The results indicate that machine learning classifiers did not always provide a better accuracy with a bigger sample size. The work in [71] also indicated that SVM’s have superior generalization capability, particularly with respect to small training sample sizes. SVM perform class separation by dividing the hyperplane optimally to maximize the distance between the two classes. Therefore, it is possible that the increase in the sample size resulted in reduced inter-class distance and caused higher classification errors. Similarly, the results in [72] showed that as the amount of training data increases, the differences in performance for different class distributions reduces because with the increase in data, the marginal class distribution becomes less in RI. In the same vein, if an increase in the sample size causes a change in the value range for the features used in training, it can reduce the accuracy of the classifiers, as well [73]. Therefore, the accuracy of SVM, RI and RF was reduced. On the other hand, there was an improvement in the accuracy of kNN, DT, NB, GBM, as well as the proposed ensemble method. An increase in the training data can lead to better predictive performance in many machine learning classifiers. The authors in [74] showed that the predictive performance is influenced strongly by sample size, and large samples produce lower predictive error in GBM. The accuracy of NB and stacking has been increased by 0.62% and 0.83%, respectively. However, if the classifiers are already showing the peak performance with a given set of data, the increase in the sample size would not yield higher performance.

### 6.4. Performance Comparison and Energy Consumption

**Figure 22:**
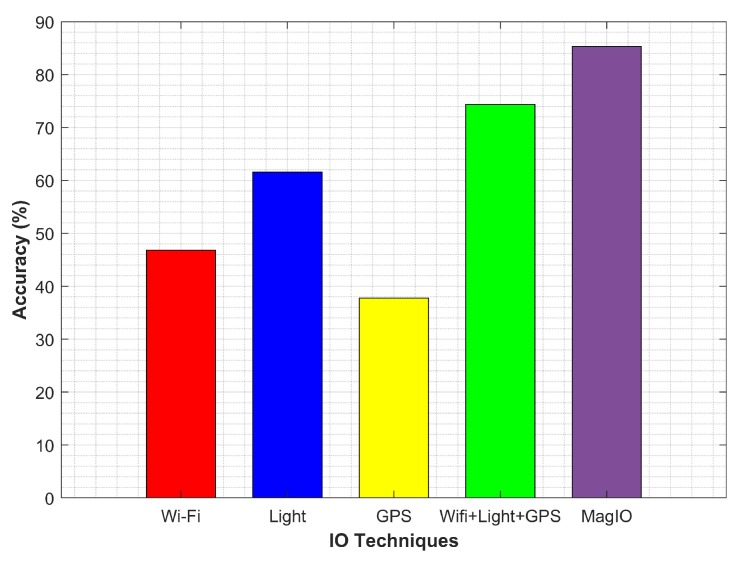
Accuracy results for IO detection with various sensors.

The performance of the proposed approach was compared against traditional technologies that are commonly used for IO problem. We collected the data for the same places using GPS, Wi-Fi and the light sensor. The collected data were used for IO detection in two ways: sensors’ individual data and fusion of all sensors data to perform IO detection. Figure 22 shows the results for the experiment.

**Figure 23:**
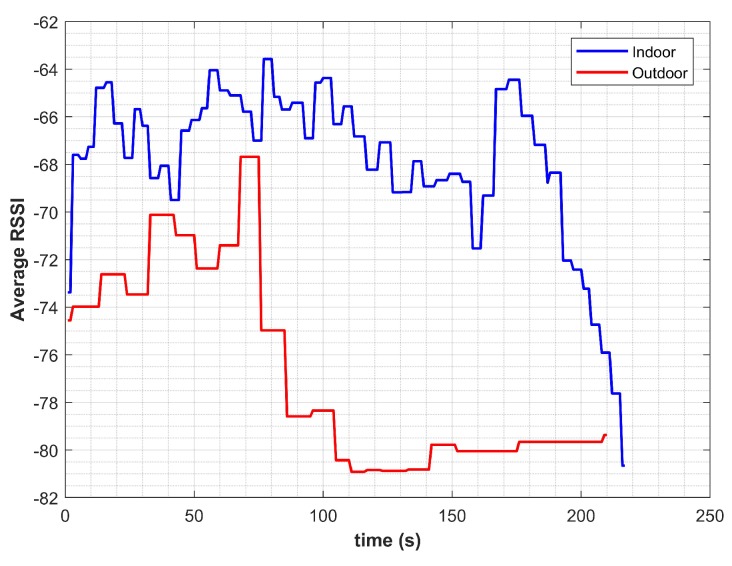
Average RSSI for indoors and outdoors.

The performance of GPS was very poor and was mainly affected when close to building entrances. It continued to report fixes, as well, when a transition was made from outdoor to the indoor environment. Additionally, it required a longer time to scan GPS signals. The performance of Wi-Fi alone was poor, as well, and it achieved an average accuracy of 46.82%. We used the number of scanned APs and average RSSI measure to discriminate IO environments. We found that Wi-Fi was not practically reliable and exhibited a similar attitude in many indoor and outdoor cases. For example, the averaged RSSI for the indoor and outdoor environment shown in Figure 23 clearly indicates that a clear threshold on RSSI was not very practical for the given scenario and so for the number of scanned APs.

**Figure 24:**
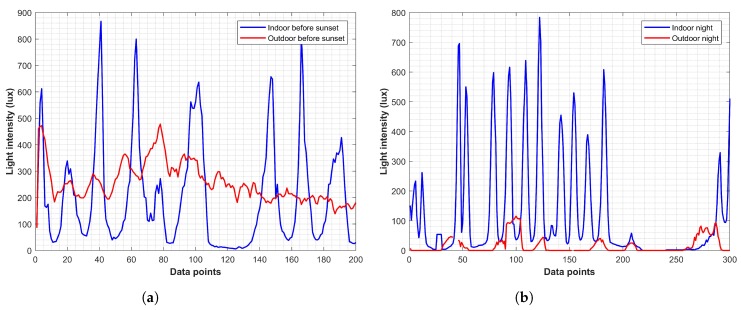
(**a**) Light intensity before sunset and (**b**) light intensity during the night.

The light sensor performed well on its own during the clear day and achieved an accuracy of 61.59%. Despite that, its performance was severely affected during various weather conditions. We conducted experiments with light sensor data collected during a different time of the day. The light sensor was used for IO detection based on the light intensity. Experimental data show that during the daytime in indoor, the light intensity was much lower than that of outdoors and vice versa during the night time. Traditionally, a threshold is used that checks for light intensity $L>T_{d}$ and decides the user environment. During the night, it checked for $L<T_{n}$ to produce the decision of the user being indoor or outdoor. The light sensors’ one limitation was its need to know the time, as well as the weather conditions. Our experiments show that before sunset, it was not possible to set a specific threshold to discriminate the IO environment. Similarly, during night, the outdoor light intensity may be very similar to the indoors, especially under bright streetlights. Data collected 30 min before sunset and 1 h after sunset shown in Figure 24 corroborated the same.

**Figure 25:**
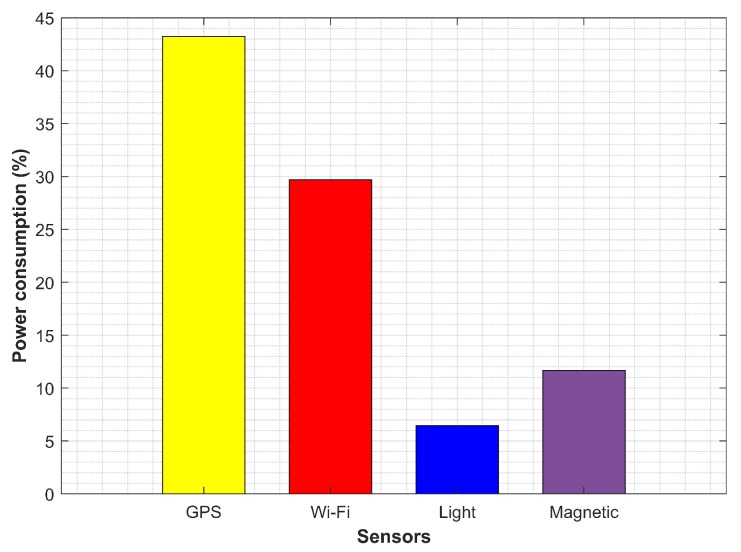
The power consumption of sensors.

The hybrid IO detection that fused the data from GPS, Wi-Fi and the light sensor could complement other sensors deficiencies and could obtain an accuracy of 70% to 80%. However, the energy consumption is to be dealt with when these sensors are combined. The average energy consumption attitude of these sensors is shown in Figure 25. GPS was the most power-hungry sensor of the smartphone and consumed 43.24% of battery, followed by Wi-Fi, which spent 29.69% of battery. MEMS-based smartphone built-in sensors including a light sensor and the magnetic sensor were less power consuming and used only 6.44% and 11.68% of the battery power, respectively. Similar power consumption results have been reported by other research works [13,75,76].

## 7. Discussion

This decade is marked by the vast development and expansion of mobile devices including tablets, wearable gadgets and smartphones. The vast proliferation of these devices has led to the development of applications that provide LBS. Location information serves as the pivotal element for such applications, which serve in tourist information, navigation, emergency rescue operations and indoor and outdoor localization. Indoor and outdoor localization systems need indoor-outdoor environment information to switch between various technologies used for localization. This switching helps to achieve better accuracy, as well as higher performance. In spite of the importance of IO (indoor-outdoor) systems, this field is not very well studied. IO systems mainly rely on GPS and cellular signal strength. However, during the last few years, smartphone sensors have been investigated for IO detection tasks. Even though, these systems rely heavily on infrastructure-based technologies, i.e., Wi-Fi, Bluetooth, cellular towers, etc., and do not take advantage of smartphone built-in sensors fully.

This research aims to leverage the smartphone sensors, especially the magnetometer, for the important task of IO detection. The magnetometer measures the strength of the ambient magnetic field, and two research works [13,23] utilized the magnetic field to assist IO detection. However, these systems used it as one unit only and relied mainly on GPS technology. This research is designed to achieve two objectives: a feasibility study on the use of magnetic field alone for IO detection and the use of machine learning to perform IO detection on magnetic data. The first task is very important as the magnetic field data are low dimensional, and the values are affected severely due to the change in the environment, as well as the smartphone orientation. Magnetic field total intensity is moderately stable, so we perform the analysis on that. A number of experiments are performed on various locations including a university campus, a shopping mall and an underground subway station. Three smartphones including the Galaxy S8, LG G6 and Galaxy Round are used for experiments. We found two groups of features during the analysis. The first group of features can individually discriminate IO environments, while the features in the second group are not a good indicator of the IO environment on their own, yet can perform classification when joined with other features. We extracted a total of 16 features and used them in seven different machine learning algorithms including DT, kNN, NB, RF, GBM, RI and SVM to perform the IO classification.

The results demonstrate that the magnetic data can potentially be used to do IO detection by using the machine learning-based techniques. Machine learning classifiers including NB and RF can achieve an accuracy of 83.26% and 78.59% respectively by taking advantage of the features extracted only from the magnetic data. Results show that NB and RF are more adaptable and noise tolerant than other techniques. Keeping in view the performance of these classifiers, an ensemble-based stacking scheme is presented that utilizes decision trees and rule induction as the base learners and naive Bayes as the ensemble classifier. This approach is able to achieve an accuracy of 85.30% on the magnetic data. The approach is compared against the performance of the light sensor-, Wi-Fi- and GPS-based IO detection and outperforms the collective performance of these sensors. GPS is limited by building entrances, as well as the canyons. Wi-Fi can practically be unreliable due to the dynamic factors of multipath and shadowing. On the other hand, the light sensor is limited by many factors including varying light conditions due to various weather conditions, bright streetlights during the night and low light conditions during sunrise and sunset. Additionally, GPS and Wi-Fi are infrastructure dependent, as well. A commercial smartphone is used to collect magnetic data, which does not require any additional infrastructure. Moreover, the magnetic field is not affected by weather conditions and is pervasive. Two scenarios that can limit the performance of classifiers with magnetic data are discussed, as well, which are the presence of large ferromagnetic materials like parking lots and concrete built over-bridges situated between closely-located buildings. The proposed approach is both accurate, as well as energy efficient.

## 8. Conclusions

This research aims to investigate the feasibility of using magnetic field data to perform IO detection using data from a magnetic sensor of a commercial smartphone. IO detection is a pivotal element for indoor localization systems to perform a smooth indoor-outdoor transition. Present systems rely heavily either on GPS or other infrastructure-dependent technologies. We aim to work out an infrastructure-free scheme leveraging only the smartphone sensors. The results prove that magnetic data can potentially be used to perform IO detection utilizing machine learning-based classification algorithms. Naive Bayes and random forest possess the capability to achieve an accuracy of 80% and higher with magnetic data alone. An ensemble-based stacking approach is presented, as well, which achieves an accuracy of 85.30% for a campus area, shopping mall and subway station using three different smartphones. Contrary to previous works that used data gathered over 8 to 10 s, we utilize magnetic data collected over 2 s only, which increases the robustness and energy efficiency. The proposed approach is robust, uses a much lesser amount of data and consumes very less power as compared to GPS- and Wi-Fi-based IO systems. We believe the findings of this work are decisively important, and the magnetic field data alone can be utilized for IO detection.

## Figures and Tables

**Table 1:** Spatial features used in classifiers.

Feature	Equation
Mean	$\mu =\frac{1}{n}\sum_{i=1}^{n}x_{i}$
Median	$\tilde{x}=\frac{x\lceil nx÷2\rceil +x\lceil nx÷2+1\rceil }{2}$
Variance	$\sigma^{2}=\frac{1}{n}\sum_{i=1}^{n}{\left(x_{i}-\mu \right)}^{2}$
Standard deviation	$\sigma =\sqrt{\frac{1}{n}\sum_{i=1}^{n}{\left(x_{i}-\mu \right)}^{2}}$
Trimmed mean	$\mu_{.25}=\frac{1}{n}\sum_{i=1}^{n}x_{i}\left(\mathrm{excludes}\;25\%\;\mathrm{high}\&\mathrm{low}\right)$
Coefficient of variance	$\hat{C_{v}}=\frac{\sigma }{\tilde{x}}$
Kurtosis	$k=\left\{\frac{n\left(n+1\right)}{\left(n-1\right)\left(n-3\right)\frac{\sum_{i=1}^{n}{\left(x_{i}-\tilde{x}\right)}^{4}}{\sigma^{4}}}\right\}-\frac{3{\left(n-1\right)}^{2}}{\left(n-2\right)\left(n-3\right)}$
Interquartile	$IQR=\frac{3}{4}\left(n+1\right)th-\frac{1}{2}\left(n+1\right)th$
Percentiles (1,10,25,50,75,99)	$i=\frac{np_{i}}{100}+0.5$
Squared deviation	$SD=\sum_{i=1}^{n}{\left(x_{i}-\tilde{x}\right)}^{2}$
Average absolute dev.	$AAD=\frac{\sum {i=1}^{n}\left|x_{i}-\tilde{x}\right|}{n}$
